# The Effects of Socioeconomic Contextual Factors on Racial Differences in Foster Care Placement Stability

**DOI:** 10.3390/ijerph22081274

**Published:** 2025-08-14

**Authors:** Leanne Heaton, William Sabol, Miranda Baumann, Arya Harison, Charlotte Goodell

**Affiliations:** 1 Chapin Hall, Chicago, IL 60606, USA; aharison@chapinhall.org (A.H.); cgoodell@chapinhall.org (C.G.); 2 Department of Criminal Justice & Criminology, Georgia State University, Atlanta, GA 30303, USA; wsabol@gsu.edu (W.S.); mbaumann1@gsu.edu (M.B.)

**Keywords:** child welfare, placement stability, socioeconomic factors, racial differences, CFSR-3

## Abstract

This study investigated how county- and state-level socioeconomic factors influence racial differences in placement stability outcomes for children in foster care. Using a sample drawn from the Adoption and Foster Care Analysis and Reporting System (AFCARS) covering 2012–2020, we employed linear mixed modeling (LMMs) to nest individual- and case-level data within counties and states. Our analysis focused on Black and White children, examining how variables such as poverty, unemployment, public welfare expenditures, residential mobility, and family structure affect the number of placement moves experienced by children. The findings indicated that Black children experience higher rates of placement instability compared to White children, although the gap narrows over time. Key factors associated with improved stability included county-administered child welfare systems and higher rates of multigenerational households and owner-occupied housing, particularly benefiting Black children. In contrast, higher levels of Supplemental Nutrition Assistance Program (SNAP) participation and increased residential mobility were linked to greater instability. The implementation of program improvement plans (PIPs) during the third round of the Child and Family Services Reviews (CFSR-3) produced mixed outcomes, with PIPs contributing to a reduction in the racial gap primarily by increasing placement moves for White children. These findings underscore the importance of analyzing data by race and incorporating broader socioeconomic contexts into child welfare improvement strategies, while also emphasizing the need for localized, context-sensitive approaches to improve placement stability.

## 1. Introduction

Racial differences in child welfare system outcomes are well-documented. From reporting to child protective services, to entry into foster care, time spent in care, and exits from care, Black children are over-represented relative to their representation in the resident population and have higher rates of involvement than their White counterparts [1,2,3,4]. Black children spend a longer time in foster care awaiting a permanent placement and experience greater placement instability than White children [5,6,7,8,9]. The number of foster care placement moves per child (or placement instability) is associated with a set of adverse outcomes for children, including delays in exiting foster care.

Building on our previous work in Heaton et al., examining one of the seven statewide outcomes—permanency in 12 months [10]—this study explores another key outcome: placement stability. Specifically, we assess how socioeconomic factors influence racial disparities on the federal placement stability measure. We examined a period in which the Children’s Bureau (CB) implemented the third round of its Child and Family Service Reviews (CFSR-3), which assessed seven statewide child welfare systems outcomes. We used multilevel models to nest individual- and case-level data within counties and nest counties within states to answer three research questions. This study makes a unique contribution to the literature by using race-specific multilevel models to examine placement stability. By nesting individual- and case-level data within counties and counties within states, our multilevel approach allows for a nuanced understanding of how structural factors at multiple levels interact with race to influence child welfare outcomes—providing critical insights to improve outcomes for children in foster care.

### 1.1. Prior Research and Knowledge Gaps

Once children are placed in foster care, child welfare agencies work to address the initial safety concerns leading to placement, followed by transitioning them to stable and permanent living situations as soon as possible. These include reunifying them with their families of origin, placing them with relatives, or finding them adoptive or other permanent homes (for example, guardianship) [11]. Having a stable placement while in care increases the likelihood of achieving permanency [12] and is essential for the child’s overall wellbeing. Stable placements promote children’s ability to form permanent, secure attachments to caretakers, healthy brain development, and successful academic achievements [13]. Regardless of children’s needs at entry, placement stability has been shown to positively affect children’s overall well-being [14].

Conversely, a greater number of placement movements (or placement instability) is associated with a reduced likelihood of family reunification or adoption [7,15], behavioral problems [16], and mental health problems [17]. Instability in placement is also linked with a larger number of adverse childhood experiences [18] and other challenges [14,15], such as loss of connections to social networks, schools, and other important contacts [17].

Most research on placement stability, however, has focused primarily on the effects of individual child, family, and case characteristics. There are some studies on the impacts of socioeconomic variables, such as family poverty or income, but they also focus on the individual level. By comparison, macro-level studies on placement stability, which either study aggregate rates at the county level or nest individual-level characteristics within counties, are rare. We discuss and draw on these prior studies to inform our approach.

### 1.2. Individual- and Case-Level Contributors to Placement Instability

At the individual and case levels, placement instability is strongly influenced by age at entry, placement setting, and reason for entry [13,17,19]. Older children at initial entry have a higher risk of placement disruption [13]. Recent estimates from the CB show that very young children more often remain in stable placements compared to children older than five years [19], and youth aged 11 to 17 years have the highest rates of instability [20,21,22].

Kinship care placements are comparatively more stable [17,23]. Non-kinship foster care is associated with higher rates of breakdown and disruption, being most pronounced in younger children [13], and placements with low-quality foster parenting are a risk factor for placement instability [13]. Child behavioral problems—particularly externalizing behaviors—predict placement disruption and the number of moves [19], even when controlling for other factors like mental and developmental disabilities [13].

There is mixed evidence for racial differences in the effects of age, race, and sex on placement stability, and most studies measure race using a categorical variable in the same model. This approach constrains the effects of the other variables included in the models to be the same between children of different racial groups. Foster et al. [8] used individual- and case-level variables but estimated separate regression models by the race of the child and then applied a statistical decomposition methodology to account for racial differences in the prevalence of risk factors associated with instability and in the relationships between these risks and instability. Their approach allowed the effects of the factors included in their models to vary between the two racial groups. They found that Black children had more placements than White children and that predictors of placement instability differed between Black and White children. For *Black children, older age, initial placement types other than kinship care*, and a *greater number of externalizing problems* were associated *with greater instability*, while for *White children*, *only the initial placement type in foster care* led to greater instability. Black–White child differences in risk factors explained most of the gap in placement instability, as well as the greater likelihood that Black vs. White children were initially placed in a foster care setting that contributed to increasing the gap. Foster et al. [8] also concluded that much of the racial disparity in placement stability remained unexplained, which suggests further examination of other drivers such as macro contextual factors.

### 1.3. Socioeconomic Factors and Placement Instability

Two lines of research characterize how socioeconomic factors affect placement stability and racial differences in stability. There are studies using individual- and case-level data and socioeconomic factors such as poverty, income, receipt of public assistance, and geographic variation as individual attributes. Others use ecological units such as counties and examine the Black–White child placement gap in the aggregate county-level placement rate in relation to measures of social disadvantage such as poverty.

The first group of studies examines individual- and case-level socioeconomic factors. Rock et al.’s [17] meta-analysis and synthesis of 58 studies conducted in the U.S., the U.K., Australia, and Canada examined both racial minority status and socioeconomic attributes of individuals and found insufficient evidence of a race effect, but a low income and poverty status of birth parents had an consistent relationship with placement stability. Helton [24] found that kinship families provided more stable placements, while simultaneously being four times as likely to fall below the federal poverty level. Kinship care families were also more likely than non-kinship families to live in disadvantaged neighborhoods [25,26] and less likely to receive foster care payments. They were more likely to receive public assistance such as Temporary Assistance to Needy Families (TANF), Supplemental Nutrition Assistance Program (SNAP) benefits, and Medicaid—benefits deemed essential for maintaining placement stability [27]. Children in rural counties were also more likely to experience more placement instability than those residing in urban areas [28].

Studies using counties as the unit of analysis are premised on the idea that the social ecology of places matters for determining the life chances of children born into different ecological environments. Social ecology is significant because it presents a set of risks and protective factors that influence child (and family) behaviors. For example, children born into communities with high poverty and lower income experience greater disadvantage in accessing high-quality schools and greater exposure to crime and violence [29], which can affect their outcomes. Economic disadvantage is associated with higher rates of reporting to child protective services, maltreatment, and racial disparities [30,31,32]. Moreover, the service agencies in less well-resourced communities have fewer means to serve needs and prevent negative outcomes, such as placement instability [33,34].

Wulczyn et al. [5] referred to the literature’s failure to distinguish between socioeconomic factors—such as poverty as an attribute of a child or family in a case—and the social ecology of poverty as the defining context in which individuals live, as a “blind spot.” In that article, as well as in Wulcyzn et al.’s [6], the authors argued that the literature on placement is relatively sparse in comparison to studies on maltreatment and other aspects of the child welfare system and that research on racial disparities in placement has largely focused on individual-level outcomes [35]. They also argued that studies on disparity in child placement rates at the county level have not examined how changes in disparity in placement rates are associated with changes in poverty and other measures of social disadvantage over time.

Using this ecological framework, Wulczyn et al. [5,6] estimated race-specific multilevel models of child placement rates as a function of race-specific measures of poverty rates and other county-level socioeconomic measures to analyze the racial placement gap cross-sectionally and over time. The separate equations approach accounted for differences in the ecological environments that Black and White children experience and allowed the effects of poverty and other measures to vary between outcomes for Black and White children. It is also consistent with other research on child welfare demonstrating the value of studying racial disparities by estimating separate models by racial group [31,35,36,37]. Wulczyn’s work also examined cross-race effects; that is, in the placement models for Black children, they included measures of White poverty rates and vice versa for the models for White children [6]. Notably, they found that Black poverty rates do not explain the Black–White placement stability gap, but the gap gets smaller with higher White poverty rates. Other studies by Heaton et al. [10], Wulczyn et al. [5,6], Drake et al. [30], and Sampson and Neil [28] also examined county covariates, such as unemployment, poverty, and homeownership, and found a relationship to child welfare outcomes. We built upon their approach in our analysis to study placement stability.

### 1.4. Federal Efforts to Improve Outcomes and the CFSR

Federal child welfare policy focuses on improving outcomes for children. In the CFSRs, the CB assesses states’ child welfare systems’ conformance with federal guidelines. The CFSRs aim to help states achieve conformity with federal requirements and assess states on seven outcomes seven systemic factors, and seven data indicators. Since 2000, the CB has implemented three rounds of CFSR, with Round 3 occurring between 2015 and 2018 [38,39]. We examined the role of CFSR-3 PIPs required under the CFSR process and implemented by states to improve the child outcome of placement stability.

For CFSR-3, placement stability was defined as the number of moves per 1000 days in foster care for children who entered during a 12-month period. It was designed to measure whether a child welfare agency ensured that children removed from their homes experienced stability while in foster care. The CB set a standard of 4.44 moves per 1000 days in care for children entering care during a 12-month period, with smaller values being more desirable [38]. For CFSR-3, no states achieved all standards [38], and states implemented PIPs to address this and other shortcomings.

### 1.5. Research Questions and Hypotheses

Building on previous research, including our own work on permanency in 12 months [10] and that of Wulczyn et al. [5,6], this study expands the evidence base on racial disparities in child welfare outcomes and offers insights into how federal and local policy mechanisms—such as PIPs—may affect equity in service delivery. Our study includes three interrelated research questions and associated hypotheses. Research Question 1 asks: *What county- and state-level socioeconomic characteristics affect placement stability for Black and White children?* We hypothesize (Hypothesis 1) that county- and state-level characteristics —such as high poverty rates, low educational attainment, and unemployment—are associated with lower placement stability for both Black and White children, but the magnitude of these associations differs by race. Research Question 2 examines whether *county- and state-level characteristics have different effects for Black and White children.* According to Hypothesis 2, socioeconomic conditions and governance structures (for example, county-administered versus state-administered systems) interact with child race to produce different placement stability outcomes. We propose that county-administered systems, being more responsive to local conditions, may either exacerbate or mitigate racial disparities depending on the local context. Finally, Research Question 3 explores whether *the program improvement plans (PIPs) implemented during CFSR-3 are associated with racial differences in achieving placement stability.* Hypothesis 3 posits that PIP implementation during CFSR-3 is associated with improved placement stability overall, but the extent of improvement varies by race, with potential reductions in racial disparities in jurisdictions that implemented more targeted or effective reforms.

## 2. Materials and Methods

### 2.1. Study Population and Data Sources

Our study population included all children and youth in the Adoption and Foster Care Analysis and Reporting System (AFCARS) covering the years 2012 through 2020. In total, AFCARS contains information for 1.365 million cases; however, to preserve confidentiality, AFCARS does not identify counties in which there are fewer than 1000 cases. Therefore, our analytic sample contained data from 114 named counties—in predominantly urban areas—over the nine years from 2012 to 2020, covering 37% of the children in AFCARS during this period. Between 25% and 30% of Black and White children in the U.S. resident population reside in identified counties. Our data included a three-year pre-CFSR-3 period (2012–2015), the CFSR-3 period (2015–2018), and a two- or three-year post-CFSR-3 period (2018–2020) when states implemented PIPs.

From AFCARS, we derived our placement stability measures and individual-level demographics, placement settings, and reasons for removal variables. We measured the race of Black and White children using Office of Management and Budget (OMB) standards for federal agencies in reporting on race and ethnicity [40]. These standards allow for flexibility in reporting; however, federal agencies report data by race for persons who select a single race and do not identify as of Hispanic origin. To maintain consistency across federal data sources, we therefore defined Black and White children as those who reported a single race and were not of Hispanic origin.

We obtained county- and state-level socioeconomic contextual variables from the U.S. Census Bureau’s American Community Survey (ACS) [41] and the Annual Survey of State and Local Government Finances (ASLGF) [42]. We used five-year ACS data for the years 2012 through 2020 and annual state-level data from the ASLGF for the years 2012–2020.

The CFSR Information Portal includes information on the year of each state’s CFSR Round 3 review and key dates on each state’s PIP. We verified the dates of PIP implementation through direct correspondence with the CB.

The State Child Abuse & Neglect Policies Database (SCAN) has data on the administrative framework status of child welfare agencies as state- versus county-administered. 

### 2.2. Measures

#### 2.2.1. Dependent Variables

Following the guidance provided in the CFSR-3 Statewide Data Indicator Data Dictionary [43], we created two dependent variables to measure placement stability. For our descriptive and county-level analysis, we calculated race-specific, county-level rates of placement stability by summing the numerators (number of moves in a 12-month period) for each child within a county, then summing the denominators (total number of days in foster care) and taking the ratio of the aggregate, county-level numerators and denominators (the results of the county-level analyses can be found in Appendix A.2). We identified deviations from the CB’s published statewide data and conducted several quality checks, including making county-level comparisons between the measures we constructed and the published data for a subset of counties, and we found negligible differences between our constructed measures and the published measures. For our second set of analyses examining individual-level outcomes, we calculated the placement stability outcome for each child. In this case, placement stability was operationalized as the number of moves a child had within a 12-month period divided by the number of days a child was in foster care during the 12-month period following the CB’s guidance.

For both measures, larger values indicate greater placement *instability* and smaller values indicate placement *stability*. For our results described below, this means that if a sign on a coefficient is positive, a variable contributed to placement *instability*. Conversely, when the sign on a coefficient is negative, that variable contributed to placement *stability*.

#### 2.2.2. Independent Variables

Individual- and case-level characteristics. We included age, race, sex, placement setting, and reasons for removal; these included reasons related to child behaviors.

County-level socioeconomic characteristics. We created both race-specific and overall county-level measures of socioeconomic characteristics using the ACS five-year data files. We used race-specific county contextual variables except when the race-specific measures were not available in the public-use data files that the U.S. Census Bureau released after conducting their disclosure-review procedures.

Building on our prior work and others, we included the following race-specific county socioeconomic measures: the **child poverty rate**, defined as the percentage of children under 18 living below the poverty line; the **unemployment rate**, measured as the percentage of the civilian labor force who were unemployed; **household income**, represented by mean per capita income; **home ownership**, calculated as the percentage of households that were owner-occupied; **residential mobility**, measured by the percentage of persons who moved within or between counties or states in the prior year; and **SNAP benefits**, indicated by the percentage of households that received SNAP benefits in the prior year.

We used two overall county-level measures of public assistance—both measure recipiency of benefits by children—and one measure of income inequality (we used the overall measures when race-specific data at the county level were not available due to Census-Bureau-imposed restrictions on the public-use data files). Such support could contribute to strengthening the capacity of families to establish stable placements; on the contrary, increases in public assistance could reflect a greater need among families that lessens their capacity to provide stable placements. These measures included the **percentage of all children receiving public welfare benefits in the past year**, the **percentage of children in female-headed households receiving public welfare benefits**, and the **Gini coefficient**, which is a measure of income inequality.

We group-mean-centered the county-level measures; each year’s estimate represents a county’s departure from the overall sample (2008–2020) average. Group mean centering removes case- and state-level variation and isolates the effect of change in county-level measures on placement stability [44,45].

#### 2.2.3. State-Level Policy and Household Characteristics

State policy. Our state-level policy variables included the following: **PIP implementation**, measured as a dummy variable equal to one (1) for the years a PIP was in effect and zero otherwise; the **percentage of state expenditure allocated to public welfare**, which encompassed direct cash payments to families, vendor payments for support services, and payments to local governments to support public and private welfare services—serving as an indicator of a state’s commitment to funding public welfare expenditures of all types; and the **administration of child welfare systems**, measured categorically as either state-administered or county-administered.

Since successful exits from foster care include placement with relatives and recent population trends show increases in the number of children living in grandparent-headed households [46,47], we used state-level measures of grandparent caregivers and multigenerational households. Grandparent caregiver households are **the percentage of grandparent-headed households within a state** where a grandparent is responsible for his or her grandchild(ren) out of all households in a state. The multigenerational households measure **is the percentage of total households in which three or more generations** live out of all households in a state.

### 2.3. Analytic Approaches and Methods

First, we described changes in the Black–White placement stability gap over time. Then, to answer our first two questions about characteristics and change in placement stability, we linked all measures across data sources using state and county Federal Information Processing Standards (FIPS) codes. We used descriptive statistics and estimated annual measures of placement stability for Black and White children in the counties in our sample.

To understand whether and how county-level contextual factors affect placement stability, we conducted several analyses using linear mixed models (LMMs), also known as mixed effects models. These models allowed us to account for the nested nature of the data and avoid producing biased standard errors. In our LMMs, children (level 1) were nested within counties (level 2) that were nested within states (level 3). The nesting, or clustering, implies that, while individual case characteristics may vary, system-involved children living in the same county are subject to the same social and institutional contexts; consequently, outcomes may cluster around county-level influences. Likewise, counties within the same state will be more like one another than counties in other states due to shared social, economic, and policy contexts. This clustering presents problems for proper hypothesis testing because the resulting random errors violate the assumptions of normality, independence, and homogeneity [48,49]. We briefly describe our modeling strategy below; however, a technical discussion of the modeling specifications can be found in Appendix A.1.

We began by estimating unconditional growth curve models, followed by a series of random slope models to test for systematic county- and state-level variation in the time trends and the effect of PIP implementation. Next, we estimated mixed effects models with random time trajectories and fixed individual-, county-, and state-level covariates. Under this approach, we allowed for variation among counties and states in their placement stability starting points and allowed for their trends to differ. We used a linear functional form for both the county- and individual-level placement stability rates, and the parameters were interpreted as effects associated with a one-unit change in independent variables.

Next, we analyzed the relationships among race-specific, county-level placement stability rates and race-specific county-level contextual variables to determine whether there are differences in these relationships by racial group. We first estimated two-level LMMs of the race-specific county-level placement stability rates, including county contextual measures for the race of the child, and then re-estimated the models, including the county measures for both racial groups.

Then, we extended our analysis to model race-specific individual-level placement stability rates, nested within counties and states. Following the approach of Heaton et al. [10], we used three-level linear mixed effects models of placement stability. Here, we simultaneously controlled for child and case characteristics and geographic areas. With the county-level rate models discussed above, we assumed that the within-county effects were constant across children. In the models of individual-level placement stability, we relaxed this assumption. We estimated separate models by race, first by including only same-race county contextual measures and then by including the contextual measures for both races.

## 3. Results

### 3.1. Descriptive and Bivariate Statistics

Our study sample was restricted to all Black and White children in foster care in named counties during the 2012–2020 federal fiscal years. These data included all children in foster care (nblack = 256,383; nwhite = 247,504) in a total of 113 counties across 39 states, yielding a total of 1232 county-year observations.

We report descriptive statistics for all measures used in our analysis and illustrate trends in placement stability over time. We test for Black–White differences in means by applying Hedges’ g, a standardized measure of the difference between two groups that accounts for differences in their sample sizes and standard deviations to calculate effect size [49]. Hedges’ g is interpreted as the standard deviation difference between groups. For example, g = 1 means that the groups differ by 1 standard deviation. Generally, g values of 0.20–0.49 are considered small effects, 0.50–0.79 medium effects, and ≥0.80 large effects [50].

We observed large effects between Black and White children across some county-level characteristics (Table 1). A larger percentage of Black than White households received SNAP benefits (28% vs. 10%), the poverty rate for Black children was more than twice that of White children (38% vs. 17%), and Black household per capita income was significantly less than White households (USD 20,491 vs. USD 33,507). Black households had comparatively lower rates of owner-occupied housing (37% vs. 66%) and much higher unemployment rates (15% vs. 7%). The main state-level characteristic showing a small racial difference was the administrative framework of the child welfare system. A larger share of Black children resided in locations within county-administered systems (38% vs. 25%).

For the individual-level and placement characteristics (Table 2), we observe a racial difference in the Black–White placement stability rates of 1.2 moves per 1000 days, with Black children averaging more moves than White children. On most of the individual-level characteristics, we did not observe statistically significant racial differences. For example, 48.2% of Black and White children were females. The average age at the most recent removal of Black children averaged 7.1 years and, while greater than the 6.2 years for White children, it was not statistically different. Black and White children had similar numbers of prior removals (1.3 vs. 1.2, respectively). On several of the current placement setting measures, the point estimates (percentage of children) appeared to differ, but none of these differences passed tests for even small effects. For example, the smaller percentage of Black than White children in pre-adoptive homes (0.6% vs 1.1%) and the percentages of Black and White children in relative foster care (35.9% vs. 40.1%) were not meaningfully different. The only individual-level characteristic that showed a small difference between the two groups was the removal reason due to the drug abuse of a parent. Fewer Black children were removed for this reason compared to White children (22% vs. 39%).

### 3.2. Racial Gaps in Placement Stability over Time

**Figure 1:**
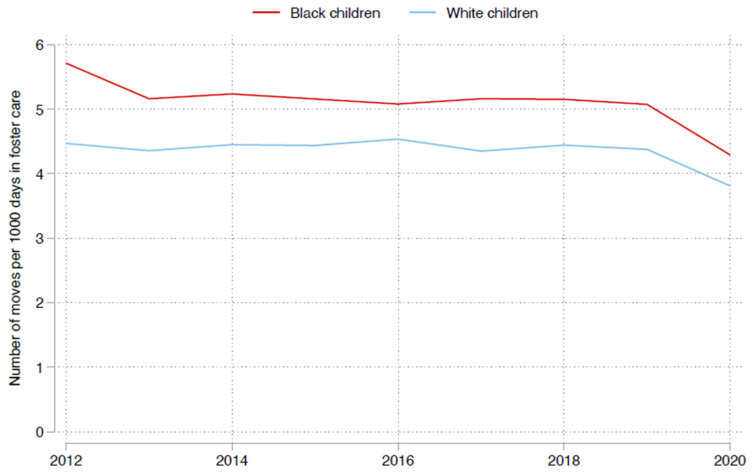
Placement rates by race of child: Placement rate is the number of moves per 1000 days in foster care.

We observed a racial gap in placement stability rates, with Black children experiencing more instability over time (Figure 1). The mean placement stability rate for Black children was 5.1 moves per 1000 days in foster care compared to 4.4 moves for White children. However, over time, placement stability improved for both Black and White children, with some convergence lessening the racial gap between the two groups. For Black children, the rates improved from 5.7 moves per 1000 days in 2012 to 4.3 in 2020 and were relatively constant between 2013 and 2019 before dropping in 2020. For White children, the rate fell from 4.5 in 2012 to 3.8 moves per 1000 days in 2020 and was also relatively constant between 2013 and 2019. Overall, the placement stability gap between Black and White children narrowed from approximately 0.8 moves per 1000 days in 2012 to approximately 0.5 moves per 1000 days in 2020.

### 3.3. Main Effects Models: Same Race Contextual Factors

To answer the first two research questions, we estimated two sets of three-level race-specific LMMs. In the first set of models, we included same-race county-level socioeconomic measures; in the second set, we included same- and other-race county-level covariates. In both sets of models, the dependent variable is the number of moves per 1000 days in foster care. The linear specification we used means that the coefficients are interpreted as the change in the number of moves per 1000 days of a one-unit change in an independent variable.

In Table 3, we report results for Black and White child models including only same-race county-level variables. In parentheses, we include the parameter estimate and its z-statistic. For Black children, the placement stability rate averaged fewer moves per 1000 days in county- vs. state-administered systems (−4.47; z = −2.63). For White children, the placement stability rate also averaged fewer moves per 1000 days in county- vs. state-administered systems (−3.03; z = −2.54). The Black–White child difference in moves was not significant.

Socioeconomic variables had mixed effects on placement stability rates for Black and White children. At the state level, increases in both state public welfare expenditures (−0.15; z = −2.82) and in the number of multigenerational households (−1.73; z = −2.92) improved placement stability for Black children. For White children, increases in public welfare expenditures led to greater instability (0.08; z = 1.98). The effects of public welfare expenditures differed between Black and White children and contributed significantly to reducing the placement stability gap by 0.2 moves per 1000 days. This reduction in the household racial gap occurred primarily because of greater improvements in placement stability for Black children.

At the county level, increases in both the percentage of children in female-headed households receiving public welfare (−0.06; z = −2.03) and in Black owner-occupied households (−0.18; z = −2.59) and decreases in Black household unemployment rates (−0.21; z = −2.91) all contributed to greater placement stability for Black children. Conversely, increases in the percentage of Black households receiving SNAP (0.18; z = 2.74) and Black households’ mobility rates (0.27; z = 4.06) led to more instability for Black children. Greater percentages of Black children under the age of 18 also contributed to more instability. For White children, only the overall percentage of children receiving public welfare was associated with improved placement stability (−0.24; z = −2.77). No other county-level measures had an impact on placement stability.

The second set of LMM results appears in Table 4, where we examined changes in findings with the inclusion of cross-race county measures. Consistent with the findings from the prior race-specific models in Table 3, county-administered child welfare systems resulted in greater placement stability for both Black and White children. Black children in county-administered systems had 4.8 fewer moves per 1000 days than those in state-administered systems (z = −2.88), while White children had three fewer moves per 1000 days (z = −2.46). The Black–White difference in the number of moves per 1000 days in foster care was not statistically significant, and the magnitude of the effects of county administration in Table 4 was comparable to those in the prior model (Table 3).

After adding the cross-race county contextual factors to the model, the effect of state public welfare expenditures (0.09; z = 2.18) increased placement moves for White children, and the effect of greater percentages of multigenerational households (−1.81; z = −3.09) reduced the number of moves (increased stability) for Black children (Table 4). These results were consistent with those in the prior models using only race-specific county-level variables. Specifically, increases in multigenerational households had a notable positive impact on improving placement stability for Black children. The larger effect of multigenerational households for Black children than for White children (difference = −1.44 fewer moves per 1000 days for Black children; z = 1.99) contributed to reducing the Black–White difference in placement stability. The total percentage in public welfare expenditures contributed to reducing the Black–White placement stability gap because of its effect on White children increasing the number of placement moves, compared to the number of moves for Black children (difference in moves = −0.19; z = −3.68).

At the county level, the number of Black children, the percentage of Black households receiving SNAP, the percentage of Black household residential mobility, Black owner-occupied housing, and Black household unemployment rates had the same effects (both in terms of direction and magnitude) as in the previous model (Table 4 vs. Table 3) (as with Table 3, here we also estimated two-level LMMs of placement stability but included both same- and cross-race county-level socioeconomic variables. We report these results in Appendix A.2, Table A2. As above, we confirm the findings of Wulczyn et al. [5,6] that White household socioeconomic status influences outcomes for Black children, but not the other way, and in comparison to Table 4, the effects of some county socioeconomic variables change in the three-level models with individual-level characteristics compared to the two-level models. Note: the variance partition coefficients/ICCs for the unconditional growth curve models are as follows: Black children: 10.03% state-level variation, 8.23% county-level variation, 81.74% variability over time (within-county); White children: 8.81% state-level variation, 6.12% county-level variation, 85.07% variability over time (within-county). However, we do not report the actual ICCs in the tables because we included random slopes. Because Stata v18’s calculation for ICCs is conditional on zero values for the random effects covariates, the “true” ICCs would have to be calculated individually for each level of each random effect (here, each panel year)). However, several cross-race factors led to greater instability for Black children; these included the percentage of White households receiving SNAP (0.43; z = 1.90), White owner-occupied housing (0.43; z = 2.90), and the White childhood poverty rate (0.38; z = 4.24). Only White household residential mobility contributed to decreasing placement moves for Black children by 5.0 moves per 1000 days (−0.50; z = −2.70). Despite this, the magnitude of the effects of these county contextual measures for White children was larger than the analogous effects for Black children.

For White children, the number of Black children in a county (0.0001; z = 3.52) and the percentage of Black households receiving SNAP (0.11; z = 3.29) increased the number of moves. The only other emergent factor contributing to placement instability for White children was the percentage of White children in poverty (0.16; z = 2.15) (none of the effects of the child-level measures changed from those reported in Table 3, so we do not discuss them here).

Three county contextual factors contributed to statistically significant changes to the placement stability gap between Black and White children: Black household residential mobility, Black owner-occupied housing, and White owner-occupied housing. Black household residential mobility and White owner-occupied housing widened the placement gap between Black and White children. For residential mobility, the Black–White child difference in moves per 1000 days was 0.35 (z = 4.26) and for White owner-occupied housing, the difference in moves per 1000 days in care was 0.47 (z = 2.51). Conversely, Black owner-occupied housing reduced the placement stability gap by −0.28 moves per 1000 days (z = −3.32) because of the positive effects for Black children.

In answering our third research question, we found that CFSR-3 PIP implementation had an effect, but it was contrary to our expectations. For White children, it was associated with greater placement instability, resulting in 0.9 more moves per 1000 days, but had no effect for Black children. However, the Black–White child difference in PIP implementation was significant. Here, PIP implementation was associated with a reduction in the racial gap in placement stability between Black and White children of 1.3 fewer moves per 1000 days for Black children. This reduction in the racial gap occurred primarily because PIP implementation was associated with an increase in placement *instability* for White children. These findings were constant across both sets of models (see Table 3 and Table 4).

## 4. Discussion

Our research expands upon Wulczyn et al. who examined the role of county-level factors and child welfare outcomes by including novel variables—such as multigenerational households and state public welfare expenditures and a three-level modeling approach [5]. As hypothesized and building on our prior exploratory work (Heaton et al. [10]), state and county socioeconomic conditions shape placement stability for Black and White children in distinct ways. When we examined the inclusion of same-race factors in our models, we found that some of these socioeconomic factors improved placement stability for Black children. At the state level, greater public welfare expenditures and a higher prevalence of multigenerational households were associated with improved stability. At the county level, higher rates of public welfare recipiency among children in female-headed households, increased Black homeownership, and reduced Black unemployment strengthened placement continuity. Conversely, elevated SNAP participation and higher residential mobility among Black households corresponded with more placement moves—a pattern that mirrors our earlier permanency findings [10] and aligns with broader research linking unemployment, food insecurity, and adverse child welfare outcomes such as maltreatment reports [51,52]. These complex interrelations underscore that indicators of economic strain and support can operate differently across contexts, highlighting the nuanced interplay of race, place, and socioeconomic conditions.

When we incorporated cross-race measures, a different pattern emerged for Black children: higher rates of SNAP participation and homeownership among White households, as well as elevated White childhood poverty, all predicted significantly more placement moves for Black children. In particular, the White childhood poverty rate was the strongest predictor of individual placement instability. This echoes Wulczyn and colleagues’ finding that county-level poverty among White children chiefly drives the Black–White gap in foster care placements and stability [5,6].

Socioeconomic factors influenced placement stability for White children as well, though findings were more mixed. In same-race models, higher state public welfare expenditures corresponded to more moves, while greater county-level public welfare recipiency among children improved stability. The county-level White childhood poverty rate also increased instability, with a larger magnitude than observed for Black children. When cross-race measures were added, higher counts of Black children and greater SNAP participation among Black households predicted more moves for White children, underscoring that placement stability reflects both local socioeconomic conditions and racial composition.

Across both races, some resource-related factors narrowed racial gaps in stability. State increases in multigenerational households and county gains in Black homeownership accelerated stability improvements for Black children, reducing the Black–White gap. Moreover, county-administered systems consistently yielded better stability—Black children experienced two to four fewer moves per 1000 days, and although the effect for White children (1.1–3 fewer moves) was not statistically significant, the larger share of cases involving Black children (38% vs. 25%) meant county administration decreased the overall gap from 17% to 12%. This likely reflects county systems’ greater policy autonomy, funding control, and responsiveness to local economic shifts, as well as their capacity to leverage community partnerships for race-specific support. While this does not imply full decentralization is the solution, it suggests that closer collaboration between state leaders and local agencies could enhance CFSR outcomes and reduce disparities.

### 4.1. Implications for CFSR Round 4 and Improving Foster Care Practice

The effects of PIP implementation were not as we expected, given CFSR’s aim to increase stability and reduce the Black–White placement gap. While PIP implementation did not affect placement stability for Black children, it led to significant increases in placement moves for White children, thereby narrowing the gap by worsening conditions for White children. A plausible explanation is that most states began their PIPs late in the study period—fewer than 30% of all cases occurred during PIP implementation, and 36 states implemented PIPs in 2019—so insufficient time may have passed to estimate their true effects reliably. Nevertheless, recognizing the influence of socioeconomic conditions on CFSR outcomes, we recommend that child welfare agencies consider Round 4 PIP foster care practice strategies that coordinate with other state and local federal programs offering resources and assistance (for example, CFSR-4 item 32 [53]).

### 4.2. Implications for Research

This study builds off our prior exploratory approach using multilevel modeling, nesting individual-level cases within counties and states to assess the role of socioeconomic factors on CFSR outcomes [10]. These findings, along with our prior findings from the three-level models, demonstrate that social ecological context cannot be ignored in studies of placement stability and other child welfare outcomes. Poverty, unemployment, and SNAP all impact CFSR outcomes, and they do so in different ways for Black and White children. They also play different roles in narrowing and widening the racial gap. Future research should examine how changes in cross-race macrolevel socioeconomic conditions affect outcomes for White and Black children, and specifically for Black children. Moreover, discussions of racial gaps in outcomes need to focus on the reasons for their changes. As we showed, worsening conditions for White children can lead to a reduction in placement differences. If one simply focused on the gap in outcomes rather than the reasons for its change, one could make the error of inferring that outcomes for Black children were improving, when this may not be the case. Future research needs to explore these complex relationships more fully, possibly by looking at the effects of within-county heterogeneity in Black and White poverty rates that could mask race-specific effects and by incorporating measures of the poverty status of the child involved in the foster care system.

Our study demonstrated that PIP implementation under CFSR-3 did not work as intended and made conditions worse for White children. A much more detailed analysis of these strategies is warranted to determine the extent to which they consisted of myopic focus on changes in child welfare practice, recruitment of specialized foster care homes, and other micro-focused efforts, rather than considering macro-level influences and policies. Based on the magnitude of the influence of White household poverty rates and the significant worsening conditions for both Black and White children, localized strategies that include antipoverty programs should also be considered in CFSR Round 4.

### 4.3. Limitations

First, we restricted our analysis to Black and White children to focus on racial disparities, prioritizing Black children due to the historical legacy of intentional separation with profound effects on over-representation. Second, our sample included only named counties in AFCARS, skewing toward urban areas and precluding meaningful analysis of Native and other rural populations, which represents a significant social justice issue given limited resources in rural settings. Third, PIP implementation coincided with the COVID-19 pandemic—most states began PIPs in late 2019 or early 2020—making it impossible to disentangle PIP effects from pandemic-related lockdowns given only one year of pandemic-era data. Fourth, our models assumed constant effects over time, yet prior work suggests non-linear and period-specific contextual influences; future studies should examine temporal variations in socioeconomic impacts. Finally, while counties serve as practical ecological units for child welfare service delivery, they may not capture neighborhood-level conditions. More granular place-based data are needed to reflect the environments where children live and to understand within-county heterogeneity in resource allocation and agency responses.

## 5. Conclusions

This study examined how county- and state-level socioeconomic factors impact placement stability for Black and White children in the foster care system. The research showed that factors such as poverty, public welfare expenditures, residential mobility, and family structure (such as multigenerational households and owner-occupied housing) affect placement stability differently by race. County-administered child welfare systems tended to have better outcomes for both groups. Additionally, while PIPs under CFSR-3 were designed to enhance stability, they unexpectedly increased placement moves for White children, contributing to a reduction in the racial gap. Overall, the findings underscore the importance of disaggregating data by race and considering broader socioeconomic contexts to inform more effective, context-sensitive child welfare improvement strategies.

## Figures and Tables

**Table 1:** Bivariate Statistics—County- and State-Level Characteristics.

Characteristic	Cases Involving Black Children		Cases Involving White Children		Diff.	Range	
	Mean%	SD	Mean%	SD	ES	Min.	Max.
State-level characteristics							
PIP implementation	23.9	0.43	25.4	0.43	0.03	0	1
CWS administration type							
State-administered	57.5	0.49	70.8	0.45	0.28 ^a^	0	1
County-administered	37.9	0.49	25.3	0.43	0.27 ^a^	0	1
Hybrid	4.7	0.21	3.9	0.19	0.04	0	1
Total public welfare expenditures	28.8	4.28	28.7	4.92	0.03	14.4	38.0
Grandparent caregiver HHs	38.9	7.61	39.2	7.87	0.04	22.8	58.2
Multigenerational HHs	3.9	1.08	3.8	1.06	0.07	1.7	8.1
General state-level characteristics							
Children receiving public welfare	32.0	8.29	29.2	6.85	0.37^a^	11.6	55.9
Children receiving public welfare in FHHs	56.9	8.69	55.1	8.41	0.21^a^	30.4	89.0
Gini index	48.82	3.32	46.71	2.38	0.73 ^b^	39.65	59.94
Race-specific county-level characteristics							
Total children	89,520	70,964.57	219,312	246,851.40	0.72 ^b^	178	1,196,651
HHs receiving SNAP	27.9	7.66	10.0	3.18	3.03 ^c^	2.2	55.8
Per capita income (USD)	20,491	3994.28	33,507	7762.29	2.12 ^c^	8748	107,888
Past year mobility	18.6	4.87	15.3	3	0.28 ^a^	7.4	40.5
Owner-occupied housing	36.9	8.83	66.0	6.88	3.26 ^c^	9.7	82.6
Unemployment rate	14.5	4.07	7.0	2.4	2.24 ^c^	0.5	30.6
Children under poverty line	38.4	7.69	17.1	5.32	3.78 ^c^	0.0%	71.9%

*Notes.* HH = household; FHH = female-headed household; ES = effect size. Effect size was computed using Hedges′ g. N = 256,383 cases involving Black children and 247,504 cases involving White children. ^a^ Small effect; ^b^ medium effect; ^c^ large effect.

**Table 2:** Bivariate Statistics—Individual Demographic and Placement Characteristics.

Characteristic	Cases Involving Black Children		Cases Involving White Children		Diff.	Range	
	Mean%	SD	Mean%	SD	ES	Min.	Max.
Placement stability (moves) (Placement stability is the overall rate of moves per 1000 days consistent with the Children’s Bureau’s definition referenced earlier in the paper.)	7.84	27.75	6.64	23.3	0.05	0	1000
Female child	48.2	0.5	48.2	0.50	0.01	0	1
Age at last removal	7.08	5.85	6.17	5.56	0.16	0	17
Total removals	1.31	0.70	1.24	0.57	0.12	1	19
Current placement setting							
Pre-adoptive home	0.6	0.08	1.1	0.10	0.05	0	1
Foster care—relative	35.9	0.48	40.1	0.49	0.09	0	1
Foster care—non-relative	40.6	0.49	40.1	0.49	0.01	0	1
Group home	6.2	0.24	5.4	0.23	0.03	0	1
Institution	8.9	0.28	5.0	0.22	0.15	0	1
Supervised independence	0.3	0.05	0.2	0.04	0.02	0	1
Runaway	1.7	0.13	0.9	0.09	0.07	0	1
Trial home visit	5.9	0.24	7.3	0.26	0.06	0	1
Removal reason(s)							
Physical abuse	16.2	0.37	10.9	0.31	0.16	0	1
Sexual abuse	2.9	0.17	3.4	0.18	0.02	0	1
Neglect	59.3	0.49	64.7	0.48	0.11	0	1
Alcohol abuse—parent	3.5	0.18	5.7	0.23	0.10	0	1
Drug abuse—parent	21.5	0.41	38.7	0.49	0.38 ^a^	0	1
Alcohol abuse—child	0.3	0.05	0.5	0.07	0.03	0	1
Drug abuse—child	1.8	0.13	2.5	0.16	0.05	0	1
Child disability	1.6	0.13	1.7	0.13	0.00	0	1
Child behavior problem	12.3	0.12	7.4	0.26	0.16	0	1
Parent death	1.0	0.10	0.8	0.09	0.02	0	1
Parent incarceration	5.4	0.23	5.8	0.23	0.02	0	1
Parent inability to cope	16.0	0.37	14.1	0.35	0.05	0	1
Abandonment	6.3	0.24	4.3	0.20	0.09	0	1
Relinquishment	1.4	0.12	0.8%	0.09	0.06	0	1
Lack of housing	8.6	0.28	10.1%	0.30	0.05	0	1

*Notes.* HH = household; GPHH = grandparent-headed household; FHH = female-headed household; ES = effect size. Effect size was computed using Hedges’ g. N = 256,383 cases involving Black children and 247,504 cases involving White children. See supplemental Table A1 in Appendix A.2 for standard deviations grouped by county and over time. ^a^ Small effect.

**Table 3:** Race-specific linear mixed model predicting individual-level placement stability for Black and White children using race-specific county covariates.

Characteristic		Black Children: Main Effects Model			White Children: Main Effects Model		
		Coefficient	Std. Error	z-Value	Coefficient	Std. Error	z-Value
Time							
Year		0.04	0.24	0.15	0.49	0.19	2.56 *
Non-linear effect of year		0.01	0.03	0.58	−0.08	0.02	−3.67 *
State-level characteristics							
PIP implementation		−0.35	0.26	−1.36	0.91	0.23	3.97 *^†^
CWS administration type							
County-administered		−4.47	1.70	−2.63 *	−3.03	1.19	−2.54 *
Hybrid-administered		−3.46	3.01	−1.15	−0.92	2.04	−0.45
% Total public welfare expenditures		−0.15	0.05	−2.82 *^†^	0.08	0.04	1.98 *^†^
% Grandparent caregiver HHs		−0.08	0.07	−1.12	0.09	0.05	1.69
% Multigenerational HHs		−1.73	0.59	−2.92 *	−0.37	0.41	−0.91
County-level characteristics							
Overall							
% Children receiving public welfare		−0.08	0.09	−0.87	−0.24	0.09	−2.77 *
% Children receiving public welfare in FHHs		−0.06	0.03	−2.03 *	−0.03	0.02	−1.23
Gini index		0.20	0.24	0.85	−0.16	0.17	−0.93
Race-specific							
Children under 18		0.00	0.00	4.20 *	0.00	0.00	−1.50
% HHs receiving SNAP		0.18	0.06	2.74 *	0.26	0.18	1.46
Per capita income (USD)		0.00	0.00	0.13	0.00	0.00	0.09
% Past year mobility		0.27	0.07	4.06 *	−0.21	0.12	−1.76
% Owner-occupied housing		−0.18	0.07	−2.59 *	0.03	0.11	0.25
Unemployment rate (%)		−0.21	0.07	−2.91 *	−0.14	0.11	−1.24
% Children under poverty line		0.04	0.04	0.93	0.12	0.07	1.67
Child characteristics							
Female child		0.14	0.11	1.30	−0.26	0.09	−2.89 *^†^
Age at last removal		0.38	0.04	10.81 *	0.32	0.03	10.67 *
Non-linear effect of age		−0.01	0.00	−5.28 *	−0.01	0.00	−6.77 *
Total removals		0.01	0.17	0.07	−0.23	0.22	−1.04
Non-linear effect of removals		0.02	0.03	0.78	0.03	0.05	0.74
Current placement setting							
Foster care—relative		1.24	0.70	1.77	1.59	0.45	3.54 *
Foster care—non-relative		4.66	0.70	6.65 *	4.70	0.45	10.46 *
Group home		7.33	0.74	9.92 *	7.19	0.50	14.45 *
Institution		7.54	0.73	10.29 *	7.24	0.50	14.40 *
Supervised independence		6.67	1.22	5.48 *	7.61	1.17	6.52 *
Runaway		7.04	0.82	8.56 *	7.80	0.67	11.58 *
Trial home visit		0.77	0.73	1.04	−0.16	0.48	−0.33
Removal reasons							
Physical abuse		1.22	0.16	7.69 *	1.62	0.15	10.55 *
Sexual abuse		0.13	0.32	0.41	0.73	0.26	2.81 *
Neglect		0.91	0.14	6.65 *	0.68	0.12	5.87 *
Alcohol abuse—parent		−0.48	0.29	−1.63	−0.49	0.20	−2.48 *
Drug abuse—parent		−0.82	0.15	−5.40 *	−1.00	0.11	−9.15 *
Alcohol abuse—child		−0.68	1.01	−0.68	1.29	0.67	1.93
Drug abuse—child		0.15	0.44	0.34	−0.33	0.31	−1.09
Child disability		−0.93	0.45	−2.04 *	−0.91	0.38	−2.38 *
Child behavior problem		−0.54	0.22	−2.43 *	−0.94	0.22	−4.33 *
Parent death		−2.53	0.54	−4.65 *^†^	−0.78	0.51	−1.54
Parent incarceration		0.37	0.24	1.51	0.49	0.20	2.46 *
Parent inability to cope		0.61	0.16	3.89 *^†^	0.00	0.14	−0.03
Abandonment		1.44	0.23	6.26 *	1.20	0.23	5.20 *
Relinquishment		−1.08	0.48	−2.26 *	−0.88	0.54	−1.65
Lack of housing		−0.06	0.19	−0.33	0.07	0.16	0.44
Intercept		16.88	4.60	3.67	−1.63	3.24	−0.50

*Notes.* * *p* < 0.05. ^†^ Mean test of differences between the two models: *p* < 0.05. Reference group (omitted category) for CWS administration type was State-administered systems. For the current placement setting categorical variable, pre-adoptive homes was the reference group (omitted category).

**Table 4:** Race-specific linear mixed model predicting individual-level placement stability for Black and White children, using same- and other-race county-level covariates.

Characteristic		Black Children: Main Effects Model			White Children: Main Effects Model		
		Coefficient	Std. Error	z-Value	Coefficient	Std. Error	z-Value
Time							
Year		0.09	0.26	0.32	0.46	0.19	2.44 *
Non-linear effect of year		0.01	0.03	0.27	−0.07	0.02	−3.30 *
State-level characteristics							
PIP implementation		−0.25	0.26	−0.98	0.93	0.23	4.03 *^†^
CWS administration type							
County-administered		−4.78	1.66	−2.88 *	−2.96	1.20	−2.46 *
Hybrid-administered		−3.44	2.96	−1.16	−0.88	2.07	−0.42
% Total public welfare expenditures		−0.10	0.05	−1.83	0.09	0.04	2.18 *^†^
% Grandparent caregiver HHs		−0.11	0.07	−1.49	0.10	0.05	1.89
% Multigenerational HHs		−1.81	0.59	−3.09 *^†^	−0.37	0.41	−0.91
County-level characteristics							
Overall							
% Children receiving public welfare		−0.08	0.09	−0.87	−0.24	0.09	−2.77 *
% Children receiving public welfare in FHHs		−0.06	0.03	−2.03 *	−0.03	0.02	−1.23
Gini index		0.20	0.24	0.85	−0.16	0.17	−0.93
Race-specific							
Black child population		0.00	0.00	3.87 *	0.00	0.00	3.52 *
White child population		0.00	0.00	−2.29 *	0.00	0.00	−1.85
% Black HH receiving SNAP		0.18	0.07	2.49 *	0.11	0.03	3.29 *
% White HH receiving SNAP		0.43	0.23	1.90	0.23	0.17	1.31
Black per capita income		0.00	0.00	0.39	0.00	0.00	0.64
White per capita income		0.00	0.00	−0.03	0.00	0.00	−0.19
% Black residential mobility		0.37	0.07	5.02 *^†^	0.02	0.04	0.40
% White residential mobility		−0.50	0.18	−2.70 *	−0.23	0.13	−1.75
Black owner-occupied housing		−0.22	0.08	−2.89 *^†^	0.06	0.04	1.64
White owner-occupied housing		0.43	0.15	2.90 *^†^	−0.04	0.11	−0.33
Black unemployment rate		−0.21	0.09	−2.37 *	−0.08	0.05	−1.58
White unemployment rate		−0.08	0.18	−0.47	−0.07	0.13	−0.53
% Black children in poverty		0.03	0.04	0.86	0.00	0.02	−0.10
% White children in poverty		0.38	0.09	4.24 *	0.16	0.07	2.15 *
Constant		16.90	4.56	3.71	−2.32	3.26	−0.71

*Notes.* Parameter estimates for child- and case-level variables are not shown; they remain unchanged from the same-race models in Table 3. * *p* < 0.05. ^†^ Mean test of differences between the two models: *p* < 0.05. Reference group (omitted category) for CWS administration type was State-administered systems. For the current placement setting categorical variable, pre-adoptive homes was the reference group (omitted category).

## Data Availability

This article examined administrative data reported to the Adoption and Foster Care Analysis and Reporting System (AFCARS). The authors received restricted access to these data and do not have permission to share them. The U.S. Census Bureau data used in this research are publicly available on the Census Bureau’s website: https://www.census.gov/data.html. (accessed on 25 May 2023).

## Abbreviations

The following abbreviations are used in this manuscript:

AFCARS	Adoption and Foster Care Analysis and Reporting System
ACS	American Community Survey
ASLGF	Annual Survey of State and Local Government Finances
CB	Children’s Bureau
CFSR	Child and Family Services Review
CWS	Child welfare system
ES	Effect size
FHH	Female-headed household
FIPS	Federal Information Processing Standards
GPHH	Grandparent-headed household
HH	Household
LMM	Linear mixed model
PIP	Program improvement plan
SCAN	State Child Abuse & Neglect Policies Database
SNAP	Supplemental Nutrition Assistance Program
TANF	Temporary Assistance to Needy Families

## Appendix A

### Appendix A.1

We used linear mixed models (LMMs) for all analyses. In both sets of analyses, we estimated random intercept models with fixed individual-, county-, and state-level slopes, and where appropriate, we used random time and program improvement plan (PIP) slopes. Under this approach, we allowed for variation among counties in their placement instability starting points, for their trends to differ, and for the effects of PIP implementation to vary.

For our analysis of county-level placement rates, we used two-level LMMs that followed the general form provided by the following three equations:

$$Y_{ij}=\;\beta_{0j}+\beta_{1j}X_{ij}+r_{ij}$$

$$\beta_{0j}=\gamma_{00}+\gamma_{01}W_{j}+u_{0j}$$

$$\beta_{1j}=\gamma_{10}+\gamma_{11}W_{j}+u_{1j}$$

where $Y_{ij}$ represents the placement instability rate outcome for a county (*i*) nested within a state (*j*); $X_{ij}$ represents predictor variables for county i nested within state unit *j*; $r_{ij}$ is the residual variance; $\beta_{0j}$ is the county-level intercept and is a function of a county-level random intercept ($\gamma_{00}$), state-level predictors $W_{j}$, and $u_{0j}$, the county residual variance; $\beta_{1j}$ is the county-level parameter for county-level predictors. The random effect $u_{1j}$ allows the effects to vary.

For our analyses that included individual-level placement outcomes, we followed the approach of Heaton et al. [10] and used three-level linear mixed models of placement stability. Our strategy encompassed the following. First, we estimated baseline fixed effects models of the form

$${PS}_{ics}=\gamma_{000}+\gamma_{001}Year+\gamma_{002}{Year}^{2}+\gamma_{003}PIP+e_{ics}+r_{0cs}+u_{00s}$$

where

*PS_ics_* is the placement stability outcome for individual case *i* in county *j* in state *k*;
Year appears as a quadratic to account for the non-linear trend in permanency rates
over time;
*γ_000_* is the random intercept, and γ_(001-3) are the coefficients for year and PIP;
*e_ics_* is the level 1 random residual; and
*r_0cs_* and *u_00k_* are the level 2 and 3 residuals, respectively.

Next, we estimated random effects models to test for systematic variation in the time trends and the effect of PIP implementation at the county and state levels. These models took the form

(A1) $$\begin{array}{cc} {PS}_{ics}=\pi_{0jk}+\pi_{1jk}{Year}_{ics}+\pi_{2jk}{{Year}^{2}}_{ics}+\pi_{3jk}{PIP}_{ics}+e_{ics} & Level\;1\\ \left\{\begin{array}{c} \pi_{0jk}=\beta_{p0k}+r_{0jk}\\ \pi_{1jk}=\beta_{p1k}+r_{1jk}\\ \pi_{2jk}=\beta_{p2k}+r_{2jk}\\ \pi_{3jk}=\beta_{p3k}+r_{3jk}\; \end{array}\right. & Level\;2\\ \left\{\begin{array}{c} \beta_{p0k}=\gamma_{pq0}+u_{00k}\\ \beta_{p1k}=\gamma_{pq1}+u_{01k}\\ \beta_{p2k}=\gamma_{pq2}+u_{02k}\\ \beta_{p3k}=\gamma_{pq3}+u_{03k} \end{array}\right. & Level\;3 \end{array}$$

where

$\pi_{0-3jk}$ are the level 1 random coefficients;
$\beta_{p0-3k}$ are the level 2 (county-level) random coefficients;
$\gamma_{pq0-3}$ are the level 3 (state-level) random coefficients; and
$r_{0-3jk}$ and $u_{00k}$ are the level 2 and 3 random effects, respectively.

Finally, we constructed mixed effects covariates models to examine the effects of individual, county, and state measures and performance on permanency in 12 months. Using a model containing random time trends at the county and state levels and random county-level PIP effects as an example, these models took the following form:

(A2) $$\begin{array}{cc} {PS}_{ics}=\;\pi_{0jk}+\sum_{p=1}^{P}\pi_{pjk}X_{ics}+e_{ics} & Level\;1\\ \pi_{0jk}=\beta_{p0k}+\sum_{q=1}^{Q}\beta_{pqk}Z_{cs}+r_{0jk}+r_{1jk}+r_{2jk} & Level\;2\\ \beta_{p0k}=\gamma_{pq0}+\sum_{s=1}^{S}\gamma_{pqs}W_{s}+u_{00k}+u_{01k}, & Level\;3 \end{array}$$

where

$\pi_{pjk}X_{ics}$ are the level 1 coefficients corresponding to individual case characteristics;
$\beta_{pqk}Z_{cs}$ are the level 2 coefficients corresponding to the county-level socioeconomic factors;
$\gamma_{pqs}W_{s}$ are the level 3 coefficients corresponding to the state-level characteristics; and
$r_{(0-2)jk}$ and $u_{0(0-1)k}$ are the level 2 and 3 random effects, respectively.

We chose a linear functional form for the placement stability rates in both the county- and individual-level rates. The parameters of our models were interpreted as effects associated with a one-unit change in independent variables.

### Appendix A.2

Table A1 and Table A2 provide regression results from two-level LMMs of county-level placement stability rates for Black and White children, where county-level aggregate outcomes are nested within states. Table A1 shows the results for models that include same-race county-level covariates, and Table A2 shows the results for models that include same-race and cross-race county-level covariates.

**Table A1:** Race-specific linear mixed model of county-level placement stability rates, with same-race county covariates.

Characteristic	Black Children			White Children		
	Coefficient	Std. Error	z-Value	Coefficient	Std. Error	z-Value
Time						
Year	0.065	0.176	0.370	−0.031	0.126	−0.250
Year-squared	−0.051	0.023	−2.240 *	−0.014	0.014	−0.950
State-level characteristics						
PIP implementation	0.077	0.347	0.220	0.352	0.184	1.910
CWS administration type						
County-administered	−1.987	0.798	−2.490 *	−1.121	0.573	−1.960 *
Hybrid-administered	−0.824	1.294	−0.640	−0.189	0.939	−0.200
General state-level characteristics						
% Total public welfare expenditures	0.016	0.041	0.390	0.041	0.028	1.430
% Grandparent caregiver HHs	−0.007	0.035	−0.200	0.035	0.025	1.380
% Multigenerational HHs	0.033	0.264	0.120	0.138	0.188	0.730
County-level characteristics						
Overall						
% Children receiving public welfare	−0.039	0.071	−0.560	−0.048	0.059	−0.810
% Children receiving public welfare in FHHs	−0.034	0.026	−1.310	−0.002	0.016	−0.120
Gini index	0.214	0.186	1.150	0.101	0.118	0.850
Race-specific						
Children under 18	0.000	0.000	1.700	0.000	0.000	0.010
% HHs receiving SNAP	0.047	0.037	1.260	0.079	0.113	0.700
Per capita income (USD)	0.000	0.000	5.410 *^†^	0.000	0.000	0.680
% Past year mobility	0.050	0.039	1.270	0.021	0.074	0.290
% Owner-occupied housing	−0.015	0.038	−0.410	−0.074	0.072	−1.020
Unemployment rate (%)	−0.051	0.044	−1.160	−0.016	0.076	−0.210
% Children under poverty line	−0.001	0.021	−0.020	0.030	0.048	0.630
Intercept	6.674	2.244	2.970	1.918	1.615	1.190

*Notes.* * *p* < 0.05. Reference group (omitted category) for CWS administration type was State-administered systems. ^†^ Mean test of differences between the two models: *p* < 0.05.

**Table A2:** Race-specific linear mixed model of county-level placement stability rates, with same- and other-race county-level covariates.

Characteristic		Black Children			White Children		
		Coefficient	Std. Error	z-Value	Coefficient	Std. Error	z-Value
Time							
Year		−0.068	0.197	−0.340	−0.020	0.125	−0.160
Year-squared		−0.037	0.024	−1.570	−0.019	0.015	−1.270
State-level characteristics							
	PIP implementation	0.113	0.344	0.330	0.354	0.183	1.930
CWS administration type							
County-administered		−1.943	0.800	−2.430 *	−1.092	0.574	−1.900
Hybrid-administered		−0.859	1.299	−0.660	−0.178	0.943	−0.190
% Total public welfare expenditures		0.005	0.041	0.120	0.038	0.028	1.360
% Grandparent caregiver HHs		−0.003	0.035	−0.080	0.036	0.025	1.430
% Multigenerational HHs		0.045	0.265	0.170	0.136	0.189	0.720
County-level characteristics							
Overall							
% Children receiving public welfare		0.005	0.041	0.120	0.038	0.028	1.360
% Children receiving public welfare in FHHs		−0.003	0.035	−0.080	0.036	0.025	1.430
Gini index		0.045	0.265	0.170	0.136	0.189	0.720
Race-specific covariates							
Black children under 18		0.000	0.000	1.860	0.000	0.000	3.370 *
% Black HHs receiving SNAP		0.086	0.040	2.180 *	0.019	0.024	0.780
Black per capita income		0.000	0.000	6.180 *	0.000	0.000	0.470
% of Black children under poverty line		0.018	0.022	0.820	−0.006	0.013	−0.470
% Black residential mobility		0.059	0.043	1.390	0.023	0.026	0.880
Black unemployment rate		0.036	0.050	0.720	−0.040	0.031	−1.260
Black owner-occupied housing		−0.006	0.039	−0.160	0.030	0.024	1.230
White children under 18		0.000	0.000	−0.140	0.000	0.000	−1.310
% White HHs receiving SNAP		0.201	0.184	1.090	0.082	0.115	0.710
White per capita income		0.000	0.000	−2.230 *	0.000	0.000	0.470
% of White children under poverty line		0.061	0.076	0.800	0.039	0.048	0.810
% White residential mobility		−0.010	0.130	−0.080	−0.003	0.082	−0.040
White unemployment rate		−0.450	0.136	−3.300 *	−0.006	0.087	−0.070
White owner-occupied housing		0.018	0.118	0.150	−0.118	0.075	−1.570
Constant		6.964	2.269	3.070	1.991	1.616	1.230

*Notes.* * *p* < 0.05. Reference group (omitted category) for CWS administration type was State-administered systems.
